# Silane coatings modified with hydroxyapatite nanoparticles to enhance the biocompatibility and corrosion resistance of a magnesium alloy

**DOI:** 10.1039/d1ra01018b

**Published:** 2021-07-29

**Authors:** Aida Nikbakht, Changiz Dehghanian, Rasoul Parichehr

**Affiliations:** School of Metallurgy and Materials Engineering, College of Engineering, University of Tehran P.O. Box 11155-4563 Tehran Iran aida.nikbakht1995@gmail.com cdehghan@ut.ac.ir rasoulparichehr@gmail.com +98 2188006076 +98 2144500749

## Abstract

The fast corrosion rate of magnesium alloys has restricted their use as biodegradable implants. Hence developing a practical approach to retard the corrosion rate of the AZ31 magnesium alloy, as well as promoting cell adhesion and proliferation is of great importance. Silane coatings were applied through dip coating, on samples pretreated in hydrofluoric acid. Samples were immersed in simulated body fluid at 37 °C, and the coating performance was assessed by electrochemical impedance spectroscopy. The coating morphologies of samples were investigated through field emission scanning electron microscopy and a cell viability/proliferation (MTT) test was performed to evaluate cellular response. A 2.2 μm-thick coating was accomplished, which increased the corrosion resistance to three orders of magnitude higher than that of the bare sample. Hydroxyapatite nanoparticles were added to the silane coating to improve biocompatibility and facilitate bone formation. Changing the concentration of hydroxyapatite nanoparticles not only helped to optimize the barrier properties of the silane coating but also ameliorated MG-63 osteoblastic cell growth. The findings showed great promise to enhance and maintain the corrosion barrier property and induce high osteoblastic differentiation by employing 1000 mg L^−1^ of hydroxyapatite nanoparticles.

## Abstract 2

Incorporation of hydroxyapatite nanoparticles in silane coatings improves both corrosion resistance and cell viability on magnesium AZ31 implants.

## Graphical abstract

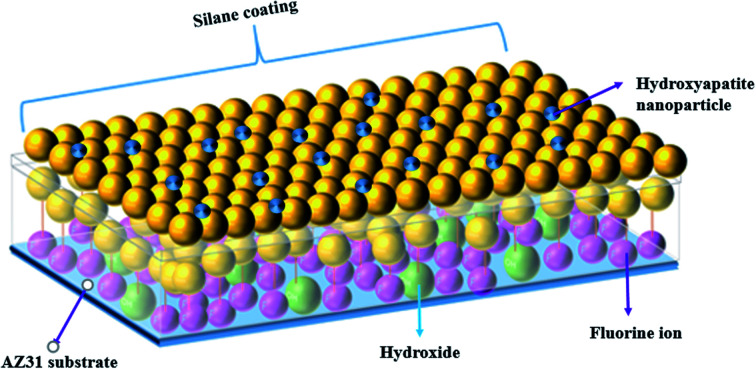

## 1. Introduction

With the advent of biodegradable materials, magnesium and a number of its alloys have drawn a great deal of attention as bioresorbable implants during the last few years.^1,2^ A good many factors contribute to making magnesium alloys a suitable substitute for permanent implants, owing to their mechanical and physical properties. They have a very similar Young's modulus and density to human bones,^3^ which eliminates the concern about stress shielding and load-bearing problems associated with titanium alloys.^4^ Magnesium stands for a non-toxic, fourth most abundant cation in the human body whose degradation can stimulate the development of hard callous at fracture sites.^5^ Magnesium alloys can be easily dissolved and absorbed in the body owing to their biodegradability property. A second surgery that may pose serious financial and medical problems can be eliminated as a result of bioabsorbability.^6,7^ Notwithstanding the fact that their application can lead to a revolutionary breakthrough, their rapid corrosion in physiological environments containing ions such as chloride has impeded their usage in the human body. AZ31 magnesium alloy corrosion is accompanied by hydrogen evolution which will postpone the healing process through forming hydrogen gas pockets.^8^

Surface pretreatments and the application of coatings have been proven to be effective methods to postpone the corrosion activity of Mg alloys. HF pretreatment, in particular, has been suggested to enhance the corrosion resistance of magnesium alloys, though it fails to render sufficient protective performance and far better results can be achieved through combining this method with polymeric coatings.^5^ Various methods, including conversion coatings,^9^ plasma spray,^10^ polymer coatings,^11^ and sol–gel processes,^12^ have been widely investigated on magnesium alloys. Among these technologies, silane coatings have proven to be an easily applied, economical approach that slows down the corrosion process and decreases the hydrogen evolution on Mg implants to a great extent.^13^ Silane coatings are organic–inorganic coatings which can retard the corrosion rate of magnesium alloys and have the potential to improve their biocompatibility.^13^ Considering that these coatings must interact favorably with bone cells, incorporation of hydroxyapatite nanoparticles is now being investigated in silane coatings. Silanes, these silicon-based, organic–inorganic materials, undergo hydrolysis in contact with water or ethanol and yield silanol groups (SiOH). Following a process of self-crosslinking, siloxane bonds (Si–O–Si) form a protective and adhesive organic layer.^14^ Silane coatings have been found to be biocompatible since they produce Si(OH)_4_ that is disposed of through the renal system;^15^ however, to the best of our knowledge, they are not reported to stimulate bone repair.

Producing a coating that can promote bone healing through producing calcium and phosphorous-rich corrosion products while presenting an acceptable corrosion resistance is undoubtedly of crucial importance. Hydroxyapatite (HA) is incorporated in different coatings on Mg implants due to properties like biodegradability, osteoconductivity and/or osteoinductivity.^16^ Since HA nanoparticles interact favorably with bone cells, they have also been used to produce hybrid/composite coatings.^17–19^

The present paper demonstrates the protective performance of silane coatings based on three different precursors. Hydroxyapatite nanoparticles (HA) were doped in different concentrations in the coatings and the corrosion, biological and morphological properties were investigated. HA nanoparticles' interaction with the silane matrix was investigated through the corrosion rate of the substrate and promotion of bone healing through producing Ca and P-rich corrosion products. In order to evaluate the biocompatibility and degradation of the applied protective coatings, the cytocompatibility of MG-63 cells was studied under *in vitro* conditions. The results showed that HA nanoparticles furnish superior bioactivity and corrosion protection in comparison with the HA0.

## 2. Materials and methods

### 2.1. Substrate preparation

An AZ31 sheet with the chemical composition presented in Table 1 was obtained from Shaanxi Taipu Rare Metal Materials Ltd, China and then cut into pieces of 40 × 20 × 5 mm^3^. The substrates were first grinded with 200 grit paper to remove the adherent oxide layer and subsequently polished with 400, 600, 800, 1000 and 1200 sand papers, rotating the samples 90° each time. Samples were ultrasonically cleaned using acetone for 6 minutes. Afterwards, they were immersed in 12% HF solution for 12 minutes and dried under an air stream as a final step in order to promote the formation of an adhesive layer which acts as a bridge and makes it possible for the further silane coating to adhere to the substrate.

**Table 1:** Chemical composition of AZ31 alloy (wt%)

Alloy	Al	Zn	Mn	Ag	Cu	Fe	Ca	Si	Mg
Mg AZ31	2.39	0.76	0.3	0.004	0.003	0.012	0.002	0.011	Base

### 2.2. Sol synthesis

The silane sols consisted of three different precursors: methyltriethoxysilane (MTES), 3-glycidoxypropyltriethoxysilane (GPTMS) and tetraethylorthosilicate (TEOS) in equal volumes (6.6% v/v) in a combination of 10% distilled water and 70% ethanol. The pH of the sols was adjusted to 4 by adding acetic acid drop by drop. The undoped silane coating, which was devoid of hydroxyapatite, was denoted as HA0. To promote biocompatibility, the three following concentrations of hydroxyapatite (HA) nanoparticles: 500, 1000 and 2000 mg L^−1^ were added and the resultant samples were denoted as HA500, HA1000 and HA2000, respectively. Hydroxyapatite nanoparticles were with diameters of below 200 nm and to make them be dispersed well in the obtained solution, an ultrasonic probe (QSONICA sonicators) was used for 30 minutes, with the solution being surrounded by 0 °C water to avoid any increase in temperature. The sols were stirred for two hours and aged for 24 hours at room temperature in order to let the precursors be hydrolyzed well and the silanol groups are formed. HF pretreated samples were dipped in the sol for 290 seconds at the immersion and withdrawal speed of 420 mm min^−1^ using a dip coater. The last step after dip coating was the evaporation of by-products, thus the samples were cured at 120 °C for 120 minutes. The oven was turned on each time as the coated samples were put inside and the temperature rose gradually from room temperature to 120 °C allowing the by-products to leave the coating.

#### 2.2.1. Surface characterization

In order to examine the morphology and dispersion of the nanoparticles before immersion and also to determine the film thickness and composition, field emission scanning electron microscope (FESEM) MIRA3TESCAN-XMU equipped with energy dispersive spectroscopy (EDS) was used. To investigate the decomposition process, corrosion products of all samples were also determined using the same apparatus after 7 days of immersion. All FESEM characterizations were done in secondary electron (SE) mode. In order to determine the film thickness, samples were embedded into epoxy resin and mold at room temperature. To prepare the sample for EDS mapping, the HA0 sample was cut by means of a guillotine machine and different layers were analyzed.

Samples were cut into 20 × 20 × 5 mm^3^ to use an ENTEGRA AFMNT- MDT atomic force microscope (AFM) in semi-contact mode, with the aim of investigating the surface roughness and providing additional information about morphology. In order to attain the roughness of surfaces, the AFM results were analyzed by Gwyddion 2.55 software.

X-ray diffraction (XRD) analyses were performed on immersed samples to determine the corrosion products, employing Cu Kα radiation in a Philips Xpert Pro PW1730 diffractometer operating at 40 kV and *λ* = 1.5405 Å. Results were analyzed *via* X'Pert High Score Plus software.

The wettability of the samples was measured using a sessile drop method (4 μL, Milli-Q water) and a contact angle meter (Veho USB Microscope 400×). Both deionized water and SBF solution were used as probe liquids to see if the SBF solution makes a difference in hydrophilicity. The results proved no staggering difference for the two liquids after repeating each test three times, hence only the results concerning deionized water are reported here. In order to ensure repeatability, the surface analyses were conducted three times on each sample.

Dynamic light scattering (DLS) technique using Cordoun Tec, Vasco, France instrument was carried out to determine the particle sizes in the suspensions containing 500, 1000 and 2000 mg L^−1^ of HA nanoparticles.

Moreover, sols with different nanoparticle concentrations were applied on coverslips and milled after the curing process to be prepared for the Fourier Transform Infrared (FTIR) spectroscopy measurements. The FTIR measurements were performed by the use of a Thermo Avatar spectrometer in the range of 400–4000 cm^−1^.

#### 2.2.2. Electrochemical techniques and corrosion products characterization

To evaluate the corrosion resistance of coated and uncoated samples, electrochemical impedance spectroscopy (EIS) measurements were carried out using a Solartron-SI 1287 potentiostat and Solarton SI 1260 frequency analyzer. The apparatus includes a three-electrode system with the sample being the working electrode (with an exposed area of 0.785 cm^2^), saturated calomel and platinum plate as reference and counter electrodes, respectively. The measuring frequency was set to range from 10^5^ Hz down to 10^−2^ Hz and the applied perturbation voltage was 10 mV. EIS tests were performed in naturally aerated simulated body fluid (SBF) pH 7.38 ± 0.1 at 37 ± 1 °C for 7 days. The SBF contained 5.403 g NaCl, 0.504 g NaHCO_3_, 0.426 g Na_2_CO_3_, 0.255 g KCl, 0.230 g K_2_HPO_4_·3H_2_O, 0.311 g MgCl_2_·6H_2_O, 0.8 g NaOH, 17.892 g HEPES, 0.293 g CaCl_2_, 0.072 g Na_2_SO_4_ in 1000 mL Milli-Q water. The ratio of sample's surface area to SBF volume was 78/5 mm^2^: 70 mL and SBF solution was prepared and refreshed every other day during the immersion period. The quantitative fitting parameters were obtained by analyzing the data using ZsimDemo 3.40D.

In order to determine the magnesium ion concentration (Mg^2+^) and pH, the bare and coated samples were immersed in SBF at 37 ± 1 °C for 7 days. The pH value was monitored at different immersion periods using a Thermo-scientific Orion 3 star pH meter and Mg^2+^ ion release was measured by an inductively coupled plasma spectrometer (Vista-MPX, Varian, ICP-OES).

#### 2.2.3. MTT assay and adhesion tests

3-(4,5-Dimethylthiazol-2-yl)-2,5-diphenyl tetrazolium bromide (MTT) assay was utilized to investigate the cell viability/proliferation on AZ31 samples. MG-63 osteoblastic cell line was utilized to investigate the cell toxicity of specimens. Cells were cultured and enzymatically dissociated from the surface using trypsin to regulate the cell concentration of the suspension to 4 × 10^4^ cells per mL and then 1 mL of the cell suspension was seeded on each of wells of the 24-well plate. In order to observe cell proliferation using FESEM, two series of coated samples were prepared to conduct the test over a period of 2 and 4 days of incubation. The culture medium of each sample was changed every other day for the samples incubated for 4 days. For the MTT assay, MTT solution was added to each well and placed in an incubator for another 4 hours to form purple crystal precipitation. Afterwards, DMSO was used to stop the reaction and dissolve the crystal precipitations. The optical density (OD) of formazan in the solution was detected by an ELYSA plate reader at a wavelength of 570 nm. For the reference purpose (100% cell viability), cells were seeded into a fresh culture medium which served as a control with the same seeding condition. The viability percentage was determined using the following formula:Viability% = (OD_s_/OD_c_) × 100In this formula, OD_s_ and OD_c_ stand for the average optical densities of the sample and the control, respectively.^20^ Each assay was performed three times.

In order to carry out the cell adhesion test, MG-63 cells were seeded and poured down the plates and onto the modified AZ31 samples, identically the same as the MTT assay. After 24 hours of immersion, samples were rinsed with phosphate-buffer saline (PBS) and prepared for fixation. Thereafter, cells were fixed using 2.5% glutaraldehyde and dehydrated using graded alcohols. Samples were then observed under a field emission scanning electron microscope.

## 3. Results and discussion

### 3.1. Surface characterization

**Fig. 1:**
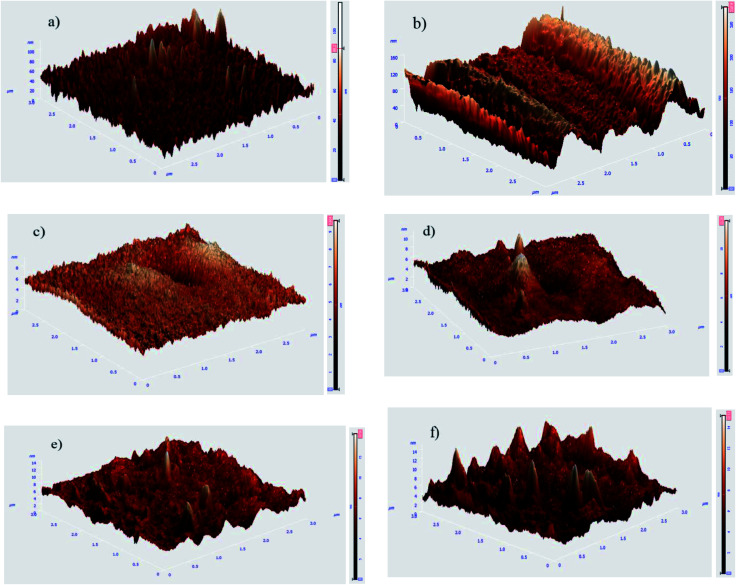
Topographical AFM images of AZ31 samples following different treatments (a) bare polished, (b) pretreated, (c) HA0, (d) HA500, (e) HA1000 and (f) HA2000 samples.

The bare, HF pretreated, undoped silane coated and three other samples, containing 500, 1000 and 2000 mg L^−1^ HA nanoparticles were tested by AFM to investigate the surface topography. Surface roughness increased as follow: HA0, HA500, HA1000, HA2000, bare and HF pretreated. It is narrated that the rougher a surface is, the more surface is available for the corrosive attack.^21^ The scans reveal a relatively rough surface with an average surface roughness (*R*_a_) of 10.06 ± 3.581 nm for the bare substrate which is associated with the polishing process. The sample pretreated with 12% HF for 12 minutes showed a rougher surface (*R*_a_ ≈ 22.54 ± 1.365 nm) with a columnar structure due to the corrosion product following the acidic pretreatment. The thickness and porosity of corrosion products are reported to determine the resultant surface roughness.^22^ Visual and microscopic inspection of the pretreated samples did not testify for a thick layer after pretreatment, hence it can be concluded that the porosity of the corrosion products was the key factor to the increase in roughness, since it renders *R*_a_ double as big. As shown in Fig. 1c, HA0 exhibited a much finer surface with a roughness of 0.6162 ± 0.3697 nm indicating a uniform thin film that is expected to be less prone to corrosion. Fig. 1d exhibits non-uniform protrusions on HA500, which have slightly increased the surface roughness to 0.6873 ± 0.4107 nm. The average surface roughness reached 0.7788 ± 0.2365 nm by incorporating 1000 mg L^−1^. The increase in *R*_a_ may be due to the increase in nanoparticle concentration, as reported in ref. 23 and 24. Incorporating higher amounts of HA nanoparticles in HA2000 revealed that the surface roughness rose to 1.107 ± 0.2572 nm. It can be concluded that the silane coating reduced the surface roughness to a large extent (less than a tenth of the bare sample) and incorporation of HA nanoparticles increased the roughness a little, which was negligible. The bare AZ31 sample exhibits a hydrophobic nature with the water contact angle being 111° and this hydrophobicity is intensified by the following HF pretreatment. Such a hydrophobic surface may impede cell adhesion and pose a problem to the tissue healing procedure. HF pretreatment followed by silane coating rendered the surface hydrophilic with the contact angle declined to 74°. Incorporating 500 mg L^−1^ of HA nanoparticles led to a 5 degree difference in contact angle, which is presumed to be due to the greater surface area provided. The increase in surface roughness provides more surface area which leads to more gas pockets getting entrapped and lower contact angles as a result, according to ref. 25 and 26. This is in agreement with our data after the incorporation of 1000 mg L^−1^ and 2000 mg L^−1^ of HA nanoparticles. Incorporating 2000 mg L^−1^ of HA nanoparticles seems to re-establish the hydrophobicity by entrapping more gas pockets. This increase in contact angle might be due to the higher polarity of this coating which can cause water uptake proneness from the viewpoint of corrosion. On the other hand, this increase in water contact angle is expected to reduce cell adhesion since the contact angle surpasses 75°.

### 3.2. Electrochemical and morphological characterization

To characterize the corrosion behavior of the bare and HA0 samples, EIS measurements were carried out as presented in Bode plots in Fig. 3a and b. Fig. 3a shows that the low-frequency impedance values of HA0 at early immersion time hits 10^6^ Ω cm^2^, which is three orders of magnitude higher than that of the bare sample. This bears the testimony that a corrosion-resistant silane coating is formed on the substrate which checks water and oxygen access to the substrate; however, the barrier effect does not remain so resistant after 7 days of immersion.^27–29^ The early decrease in impedance modulus at low frequencies can be assigned to the deterioration of coating barrier properties as a result of electrolyte uptake which is not favorable after 7 days of immersion; this result is consistent with previous findings.^5,30^ At the early immersion durations, two time constants are noticeable in Fig. 3b, the high-frequency time constant, emerging at 10^4^ Hz, is associated with the silane coating resistance and capacitance. As time passes the time constant lying at high frequencies (10^4^ Hz) moves towards the medium frequencies (10^3^ Hz), implying that the silane coating is not so resistant as before. The low-frequency time constant lying at almost 0.1 Hz at the first hour of immersion is related to the capacitance of the double layer on the metal/electrolyte interface. After 7 days of immersion, the two time constants seem to be inextricable, which suggests that the silane coating has lost its barrier property.^31^ According to the phase angles in Fig. 3b, the bare sample reveals positive angles after an hour of immersion in low frequencies (between 10^−1^ and 10^−2^), which is eloquent of an inductive loop. The inductive loop is indicative of adsorption taking place on the bare AZ31 much sooner than HA0. The early adsorption puts forth the fact that the accumulation of corrosion products hampers general corrosion and transforms it to pitting corrosion through a depressing galvanic effect between the two phases.^32,33^ HA0 tended to shift to positive phase angles and reveal the inductive loop after longer hours of immersion since corrosion is postponed and it might be due to the relaxation of the adsorbed species.^34^ A glance at the overall reaction for magnesium corrosion (Mg + 2H_2_O = Mg^2+^ + 2OH^−^ + H_2_) suggests that a rapid degradation process conduces to an elevated release of hydrogen gas and an increase in the local pH value, both of which make the environment incompatible for the surrounding tissues.^13^ The rise in the pH can impede cell adhesion on the implant surface and induce toxicity which is unfavorable. More seriously, the mechanical strength of the implant might be lost prior to the completion of the healing process. As a result, a more corrosion-resistant coating with lower level of alkalization and hydrogen emission is considered to ameliorate the case by laying the groundwork for the osteoblasts to grow on and instead of the implant.^35^

**Fig. 2:**
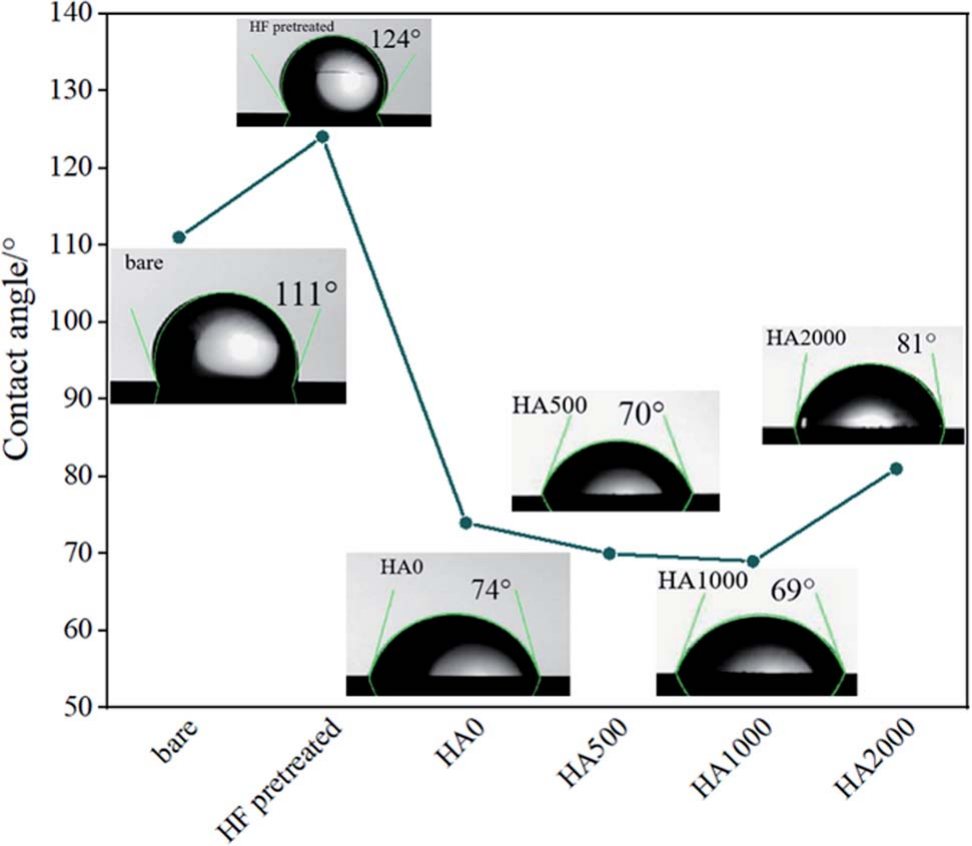
Contact angle measurements of the bare, HF pretreated, HA0, HA500, HA1000 and HA2000 samples.

**Fig. 3:**
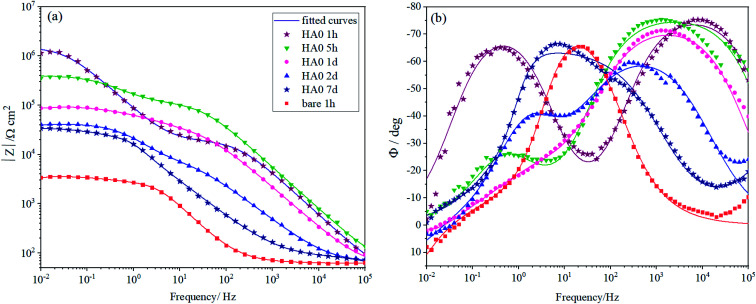
(a and b) EIS spectra for bare and HA0 during immersion in SBF.

**Fig. 4:**
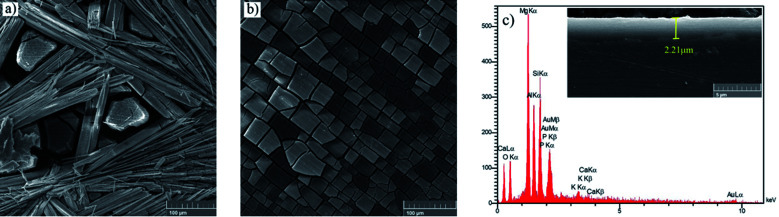
Morphology of (a) bare, (b) HA0 after a week of immersion in SBF, (c) cross-sectional image and EDS analysis of HA0 before immersion.

Fig. 4a depicts the surface morphology of the bare and HA0 samples after 7 days of immersion in SBF at 37 ± 1 °C. The bare sample was covered by the corrosion products, which seem to be adhesive and protective to some extent since the bare sample demonstrated a higher impedance modulus at low frequencies after 7 days of immersion (the results are not shown here). The presence of fiber-like corrosion products on the surface of the bare sample indicated a high corrosion rate taking place on the first days of immersion, which is accompanied by abundant magnesium ion release and a dramatic increase in pH values. Fig. 4b demonstrates a homogeneous morphology for HA0, which is replete with superficial cracks. These micro-cracks facilitate the penetration of electrolyte to the coating/substrate interface and conduce to inadequacy in coating resistance.^36^ The absence of fibrous corrosion products on the HA0 is indicative of the fact that the substrate is not involved in the corrosion process as much as the bare sample. Visual inspection of the bare and HA0 confirmed the existence of pits on the surface which is in accordance with inductive loops and positive phase angles observed in low frequencies in impedance spectroscopy shown in Fig. 3b. The cross-sectional SEM image of HA0 did not reveal any defects such as micro-cracks or micro-pores. The coating revealed a homogeneous thickness of 2.21 ± 0.32 μm, which was well adherent to the substrate as a distinguishable border was not recognized in Fig. 4c. The EDS analysis from the cross-section of HA0 before immersion (Fig. 4c) revealed a composition with a high ratio of Si and O, indicating that the curing time and temperature were sufficient for the cross-linking process to take place.

Modification of silane coatings with different nanoparticles and/or corrosion inhibitors seems to improve the coatings' protective properties.^11,37^ Since hydroxyapatite nanoparticles enjoy the same composition as the inorganic part of human bone, they are biocompatible. HA nanoparticle properties, including a high surface area to volume ratio, an ultrafine structure similar to biological apatite and grain size, are expected to introduce gratifying functionalities.^18^ HA nanoparticles have previously proven to act as corrosion inhibitors because of their buffering effect and reducing potential gradients between anodes and cathodes.^38,39^ Zomorodian *et al.*^18^ also investigated the effect of incorporation of hydroxyapatite in polymeric composite coatings and HA amounts lower than 5% were found to enhance the corrosion resistance while keeping the biocompatibility. In the present study, HA nanoparticles at different concentrations were used, aiming at progress in biocompatibility and corrosion resistance of the silane-coated samples. Particle sizing was carried out by DLS for all HA nanoparticle-containing suspensions. As it is seen in Table 2, the nanoparticle mean size increases to almost double as big (1.823 times bigger) by increasing the suspension concentration from 500 mg L^−1^ to 2000 mg L^−1^. Particle size increase and inhomogeneity in particle size distribution are inevitable to a great extent when higher amounts of the HA nanoparticles are incorporated. Inhomogeneous particle distribution can create tension in the coating, which results in the formation of preferential pathways for electrolyte penetration.^18^ Since there are only a few articles reporting HA modified silane coatings, the effect of time and power in sonication has not been investigated. In the current study, samples were ultrasonically agitated for 30 minutes, however, it is reported that longer durations and higher rates of agitation lead to the destruction of agglomerates. This is due to the shear stress exerted on agglomerates, overcoming the van der Waals force, which holds the particles together.^15^

**Table 2:** Distribution of HA nanoparticles with DLS method

Sample	Distribution statistics		
	HA500	HA1000	HA2000
Mean size (nm)	344.1	454.4	627.5
Mode (nm)	340.1	443.8	615.8

**Fig. 5:**
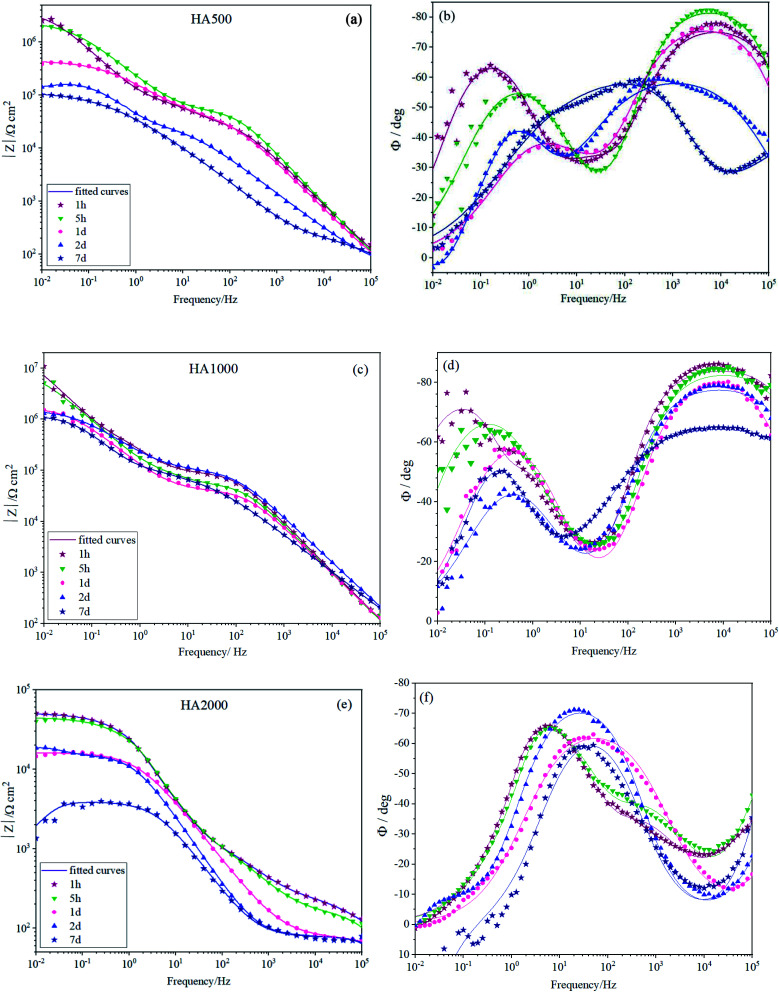
EIS spectra of (a and b) HA500, (c and d) HA1000 and (e and f) HA2000 immersed in SBF for different durations.

Fig. 5 depicts the Bode plots for the coated alloys containing nanoparticles with different concentrations varying from 500 mg L^−1^ (HA500) to 2000 mg L^−1^ (HA2000). It is worth mentioning that relatively resistant coatings were seen in Fig. 5a and b, and employing 500 mg L^−1^ HA nanoparticles did not interfere with the coating formation. Fig. 5b shows the time constant lying at 10^4^ Hz, indicating that the covalent bonds are successfully formed since the time constant at high frequency is attributed to the silane film. HA500 seemed to be effective in maintaining the silane coating's barrier properties since after 7 days of immersion impedance modulus at low frequencies were higher than that of the HA0 sample. The concentration of the nanoparticles was increased to see whether it would culminate in a more resistant coating by passing time. As seen in Fig. 5c and d, the impedance modulus at low frequencies hit 10^7^ Ω cm^2^ for HA1000, which demonstrates a coating with an order of magnitude more resistant to corrosion than that of the HA0 (10^6^ Ω cm^2^) at the early immersion durations. This suggests that an optimum amount of HA nanoparticles can improve the barrier effects of silane coating. The accomplished resistance was higher in comparison to the ones reported.^40^ After a week of immersion, HA1000 exhibited almost the same resistance as HA0 after an hour of immersion. There was no sign of an inductive loop in HA1000 and HA500 at any immersion duration, which reiterated the elimination of pitting corrosion as long as 7 days. HA1000 was expected to be an aid to cell growth and not to release much hydrogen since there was an insignificant decrease in the resistance. The high-frequency time constant appearing at frequencies around 10^4^ Hz in the case of HA0, HA500 and HA1000 (see Fig. 5b and d), does not show up in HA2000 (see Fig. 5e) showing that a resistant silane coating has not formed on the Mg(OH)_*x*_F_2−*x*_ layer. This was in line with the visual inspection of HA1000, which did not reveal a glass-like coating. It can be concluded that the excessive amount of HA nanoparticles hampered condensation reaction and left many silanol groups uncondensed.^41^ The presence of uncondensed silanol groups in the coating leads to the formation of a less reticulated network that does not possess good physical barrier properties against the aggressive solution. This underlines the fact that the achieved resistance (5 × 10^4^ Ω cm^2^) was not accomplished by silane coating but most likely by the process of pretreatment in HF.

**Fig. 6:**
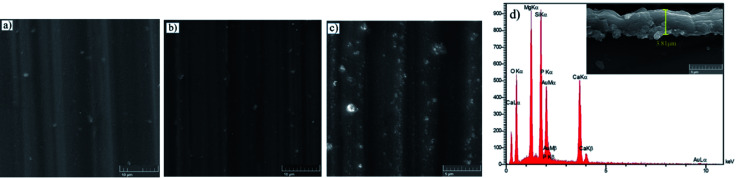
FESEM micrographs of (a) HA500, (b) HA1000, (c) HA2000 before immersion and (d) cross sectional secondary image of HA1000 before immersion.

**Fig. 7:**
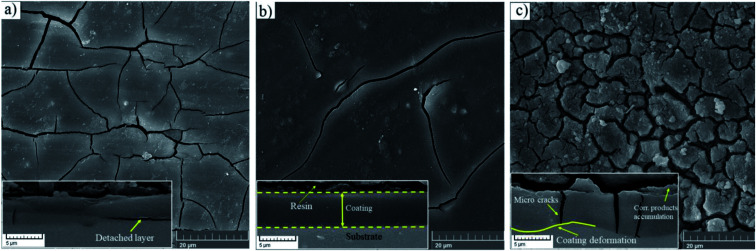
Morphology of corrosion products after a week of immersion in SBF at 37 ± 1 °C (a) HA500, (b) HA1000, (c) HA2000, the inset underneath each morphology depicts the cross sectional view.

The top views of the HA doped samples before immersion are shown in Fig. 6. The as-prepared HA500 evinced a homogeneous, crack-free surface with observable polish marks but no sign of nanoparticle agglomeration. Moreover, the uniform dispersion of HA nanoparticles is illustrated in Fig. 6b which ensures the uniform corrosion and biological properties. Fig. 6c clearly shows that HA nanoparticles have a sphere-like morphology which is more favorable for MG-63 osteoblastic cells than that of the rod-like shape. It is reported that sphere-like nanoparticles are more likely to provide a well-organized surface which is beneficial for filopodia protrusion.^42,43^ It can be inferred that the increase of HA nanoparticle concentration to 1000 mg L^−1^ may lead to bigger particle sizes (454.4 nm), but the agglomerated particles do not adversely affect the corrosion performance of the coating. The cross-section of the HA nanoparticle-containing samples was investigated to examine the impact of HA nanoparticle doping on the thickness and homogeneity of the coating. The increase in coating thickness was observed in the systems similar to our work and reported to be due to an increase in silane sol viscosity.^18^Fig. 6d depicts the cross-section of HA1000, with the coating thickness being 3.81 μm which is eloquent of a 1.6 μm increase in comparison to that of HA0. When nanoparticles are incorporated, an increase in the coating thickness is not necessarily conducive to higher corrosion performance. Surface FESEM images of the HA nanoparticle-containing samples after 7 days of immersion are shown in Fig. 7. A comparison between surface morphologies of the corroded HA0 (Fig. 4b) and HA500 samples (Fig. 7a) reveals that incorporation of HA nanoparticles reduces the cracks to a great extent. Although a number of randomly scattered cracks are visible on the HA500 top coat in Fig. 7a, employing HA nanoparticles seems to ameliorate the coating integrity after 7 days of immersion. Higher concentrations of HA nanoparticles are expected to lead to fewer cracks on the surfaces since HA1000 revealed a higher corrosion resistance. No defects are seen in Fig. 6b, which depicts HA1000 upon coating, indicating that nanoparticles are not added to an excessive amount. HA nanoparticles seem to reduce electrolyte permeation into the coating and the substrate exposure to the electrolyte. Fewer cracks were seen in Fig. 7b, indicating better corrosion protection which was in agreement with EIS results. As a matter of fact, incorporating 2000 mg L^−1^ of HA nanoparticles worsened the protective properties of the coating and let more magnesium ions from the substrate be detached. In the case of HA2000, the coating's barrier properties deteriorate faster than the other samples, this process most probably starts with electrolyte penetration through pits, which shows up as an inductance in Fig. 9c. The fast corrosion process is accompanied by accumulation and deposition of corrosion products which exert mechanical stress on the silane coating and lead to crack formation and coating deformation visible in the inset in Fig. 7c. As shown in Table 3, the chemical composition of corrosion products on all samples was investigated through EDS analysis. The presence of Mg and O was confirmed in corrosion products on all samples after 7 days. This puts forth the fact that Mg oxides and/or hydroxides are formed as primary corrosion products.^44^ Magnesium percentage shows a decreasing trend for the samples with HA nanoparticles incorporated, this suggests that fewer magnesium ions were released by immersion in SBF after 7 days of immersion; however, this is not an ever-decreasing trend, and HA2000 proves the reverse (with Mg atomic percent being 22.79). Ca was detected for the coated samples with HA-incorporated nanoparticles; however, the Ca/P ratio was not satisfactory. No Ca was detected for the HA0 and bare samples, which underlines the fact that Ca was not coming from the SBF solution to a calculable extent by EDS analysis, but it was coming from the doped HA nanoparticles in the modified samples. The corrosion products on HA containing samples became richer of Ca and P as the concentrations of the nanoparticles were increased. As reported in Table 3, the Ca/P ratio for HA1000 was 1.464, which was very close to that of stoichiometric hydroxyapatite, whose Ca/P ratio is 1.67. It is reported that hydroxyapatite crystals precipitated from supersaturated aqueous solutions are non-stoichiometric in most cases. This is because of the defects like vacancies present in the crystal lattice being introduced into the precipitating system.^45^ The Ca/P ratio in HA1000 reiterates the fact that the coating is highly likely to promote bone formation through expediting and facilitating the process of bone repair.

**Table 3:** Surface chemical compositions of all samples after 7 days of immersion measured by EDS in atomic percent

Element	Mg (at%)	Al (at%)	Si (at%)	Ca (at%)	P (at%)	O (at%)	Ca/P
Bare	15.86	0.48	0.12	—	26.18	47.01	0
HA0	16.26	1.31	45.70	—	11.66	36.73	0
HA500	12.90	1.55	10.32	8.57	6.05	60.61	1.416
HA1000	7.03	1.48	14.21	7.98	5.45	63.84	1.464
HA2000	22.79	1.37	12.55	7.21	4.76	51.33	1.514

**Fig. 8:**
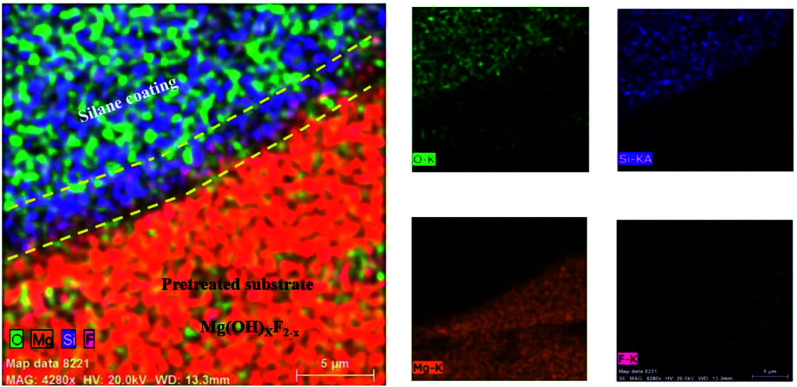
Elemental mapping images for the silane-coated part of the substrate and the area where the coating was detached.

The adhesion of the HF pretreated/silane-based composite coating was determined according to ASTM D3359. All samples were rated 5B, which corresponds to the best ASTM rating. No sign of detachment was observed on any of the samples, proving that the addition of HA nanoparticles at any concentration has not apparently interfered with adhesion to the substrate. It is reported that the HF treatment on magnesium alloys leads to the formation of a compound with the general formula of Mg(OH)_*x*_F_2−*x*_, which testifies for the simultaneous presence of magnesium hydroxide and fluoride.^46^ Also, according to Ji *et al.* hydrogen bonding is formed between F^−^ in MgF_2_ and hydroxyl groups.^69^ It can be concluded that the satisfactory adhesion of silane coating to magnesium substrate is due to the formation of (1) hydrogen bonding between silane hydroxyl groups and the fluoride formed the substrate and (2) the Si–O–Mg bonds formed as a result of the reaction between silane hydroxyl groups and magnesium hydroxide. In order to investigate how this interlayer is formed, the HA0 sample was cut by a guillotine machine and a part of the coating was detached inevitably through the cutting process. Fig. 8 illustrates the coated part of the substrate which conjoins the part with the coating detached. The lower part in Fig. 8, where the coating has been detached is replete with fluorine and oxygen. The presence of F after the removal of silane coating testifies for the great adhesion of the thin film attained by HF pretreatment. This film which is a combination of magnesium hydroxide and fluoride (Mg(OH)_*x*_F_2−*x*_), makes it possible for the Si–O–Mg bonds to be formed. The part bounded by silane coating and the substrate reveals the mechanism through which Si–O–Mg bonds are formed, since the coating has been partly detached and both of the F and Si are seen.

### 3.3. Corrosion modeling

**Fig. 9:**
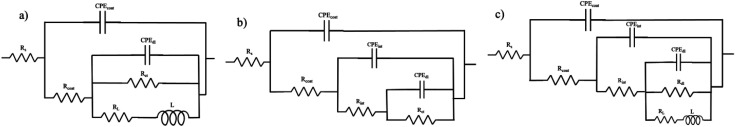
Equivalent electrical circuits used to fit EIS data. (a) HA0, (b) HA500 and HA1000, (c) HA2000.

**Fig. 10:**
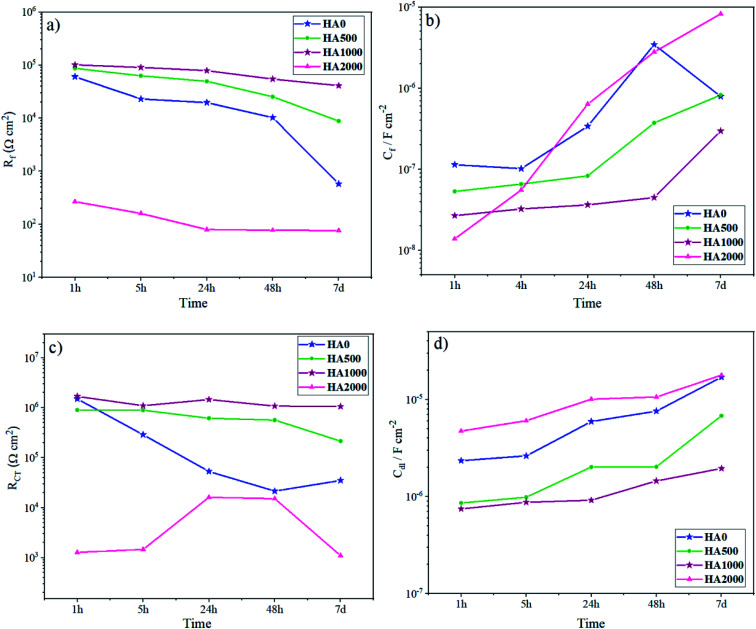
Evolution of the (a) silane coatings' resistance, (b) silane coatings' capacitance, (c) charge transfer resistance and (d) CPE parameter.

In order to interpret the EIS data in detail, numerical simulation was employed using equivalent circuit models. The evolution of the resistance and capacitance for HA0 and HA1000 samples, determined by fitting, are presented in Fig. 9. In this simulation, constant phase element (CPE) was used to describe a non-ideal capacitance instead of an ideal capacitor. Factors like slow adsorption reactions, surface heterogeneities and roughness, a non-uniform potential and current density are among the involved factors in a non-ideal capacitive behavior.^47^ For HA0, a two-time constant electrical equivalent circuit (EEC) was used to fit the impedance data (Fig. 9a). *R*_coat_ and *R*_ct_ denote the electrolyte resistance in the coating pores and charge transfer resistance, respectively. CPE_coat_ accounts for the capacitive properties of the silane coating, CPE_dl_ for double-layer capacitance, which reveals corrosion activity present at the coating/substrate interface and *L* for inductance which appears at low frequencies. *C*_int_ corresponds to the capacitance associated with this interfacial layer, and R_int_ accounts for the resistance of the pores in the interlayer. Since different circuit models were used to simulate the coatings, a combination of *R*_f_ and *C*_f_, which corresponds to the overall resistance and capacitance of the coating response, was employed to evaluate the barrier effects of the protective layer. *R*_f_ attained identical values for all samples except for HA2000 at the early stages of immersion (Fig. 10a). In the case of HA0 *R*_f_ starts to decrease much faster than the HA500 and HA1000, which puts forward the fact that water uptake was taking place through the outer layer. Though the outer layer of the silane coating in the case of HA0 did not remain protective enough after 168 h (7 days), the inner layer acted as a barrier for a longer period. The EIS spectra for HA0 in Fig. 3b revealed positive phase angles during the first and second day of immersion, which represent an inductive loop in the Nyquist plot (not reported here). The inductive loop at low frequencies may be attributed to the relaxation of adsorbed species that already covered the surface during the first 24 hours of immersion and culminated in the substrate being prone to localized corrosion.^48^ Modifying the silane coating with HA nanoparticles seemed to ameliorate the protective properties of the coating since *R*_f_ hits 41 kΩ cm^2^ after 168 h of immersion, which was over 100 times higher than that of HA0. An interlayer appearing in the equivalent circuit for HA1000 and HA2000 (see Fig. 9b and c) correspond to the interfacial layer replete with Si–O–Si and Si–O–Mg bonds. These bonds provide good adhesion properties to the substrate, delaying the exposure of the substrate to a corrosive environment. Though these bonds were already formed in HA0, they seem to form an independent layer in the HA1000 and HA2000, which shows up in Fig. 9b and c. Inductance does not appear for HA500 and HA1000 with increasing the immersion time, which means that local corrosion in general and pitting corrosion, in particular, have been controlled. The slower increase in *C*_f_ values for HA1000 also underlined the postponed deterioration in barrier properties.

### 3.4. Structural analysis – by Fourier transform spectroscopy (FTIR)

**Fig. 11:**
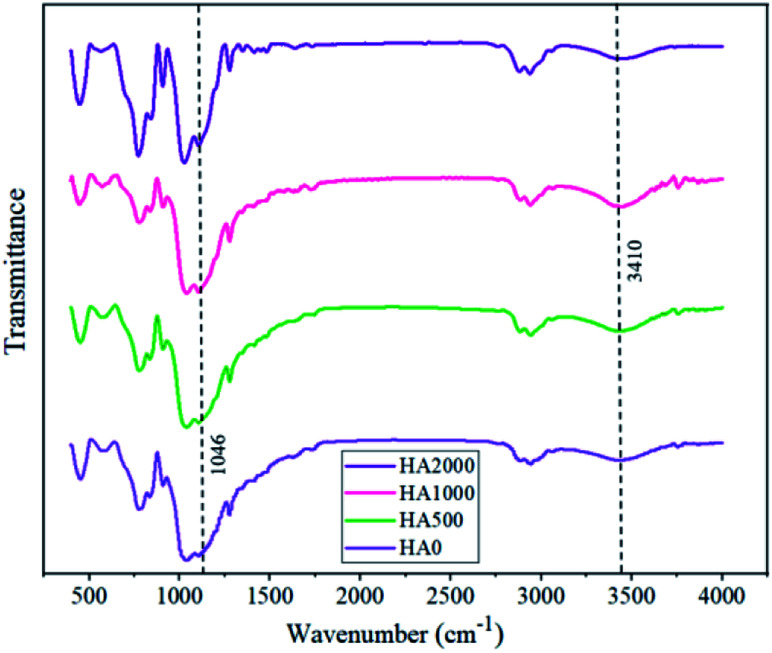
The effect of hydroxyapatite nanoparticles on the FTIR spectra of silane coating powder.

Superior corrosion protection in the presence of specific concentrations of HA nanoparticles might be inferred from Fig. 11, showing the FTIR spectra for all samples. The peaks that appear at 1046 and 3410 are ascribed to the Si–O–Si bonds and Si–OH, respectively. Considering the fact that silanol groups are consumed in the crosslinking during curing and form siloxane network, lower ratios of Si–O–Si to Si–OH peak length indicate higher cross-linkage and more reticulated networks. According to Table 4, the FTIR spectrum of HA500 and HA1000 revealed lower ratios of Si–O–Si to Si–OH peak length in comparison with that of the HA0. This is due to the reaction of silanol groups with the surface of nanoparticles which are probable to make the film denser.^49^ Moreover, the increase in hydroxyapatite concentration led to a less reticulated network in the case of HA2000 with a 0.283 ratio. There are two reasons for the adverse effect high nanoparticle concentrations have on condensation: (1) excessive amounts of nanoparticles fail to disperse homogeneously^50^ (2) some hydroxyapatite nanoparticles remain uncondensed and do not contribute to crosslinking, leading to the formation of active sites.^41^

**Table 4:** The ratio of Si–OH to Si–O–Si peak in FTIR spectra

Sample name	HA0	HA500	HA1000	HA2000
Si–OH to Si–O–Si peak ratio	0.180	0.170	0.121	0.283

### 3.5. Magnesium ion release and pH change

**Fig. 12:**
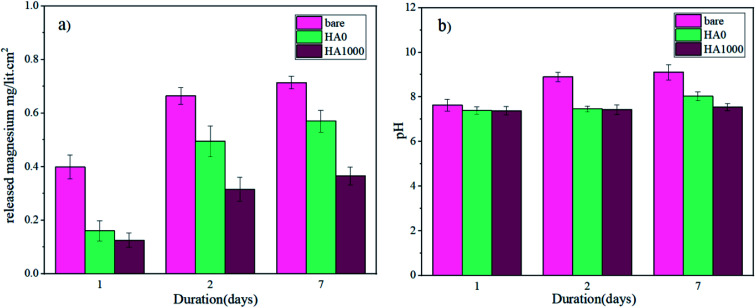
(a) ICP-OES measurement of released Mg ions and (b) pH changes over a period of 7 days.

Mg^2+^ ion concentrations for the bare, HA0 and HA1000 samples were analyzed by ICP-OES, and the results are shown in Fig. 12a. The Mg^2+^ ion concentration for all samples rose to different levels, suggesting that corrosion is occurring at different rates. Mg^2+^ ion concentration after 7 days of immersion hit 0.714, 0.57 and 0.365 mg L^−1^ cm^−2^ for the bare, HA0 and HA1000 respectively. This puts forward the fact that silane coating has retarded the corrosion process, and hydroxyapatite nanoparticles have culminated in a conspicuous decrease in the corrosion of the substrate. The initial increase in Mg^2+^ ion concentration for the bare sample was due to the formation of fibrous corrosion products. The slower increase in Mg^2+^ ion concentration for the bare sample after 7 days of immersion corroborates that the fibrous corrosion products were relatively protective. For the HA0, only a slight increase was seen in Mg^2+^ ion concentration after 7 days of immersion in comparison with the 2^nd^ day. This suggests the fact that Mg^2+^ ion release, which is indicative of the substrate being exposed to the electrolyte, started on day 2. In fact, the HA0 coating did not remain protective enough and electrolyte permeated through this coating on the second day of immersion. Furthermore, the bulk pH changes recorded over a period of 7 days are also available in Fig. 12b. Marked changes in pH and Mg ion release are doubtlessly accompanied by hydrogen evolution which can impede the cell growth and attachment.^51^ In addition, managing the pH values is of great importance since it is an important variable inflicting cell viability.^52^ The initial pH of the SBF upon immersion was 7.28 and it can be seen that the pH value has increased for all samples. The pH value for HA1000 reached 7.54 after 7 days of immersion which seems to be an aid to cell attachment, while the same parameter was 9.11 and 8.03 for the bare and HA0 samples, respectively. The sudden increase in pH and Mg ion concentrations in the bare sample, can be considered a hindrance for cell growth and may conduce to inflammation. In case of the bare sample Cl^−^ in SBF solution seems to transform Mg(OH)_2_ to soluble Mg(Cl)_2_ which later dissolves to Mg^2+^ and 2Cl^−^, being conducive to an increase in hydroxide (OH^−^) ions on the surface of the sample.^53–55^

### 3.6. XRD analysis

**Fig. 13:**
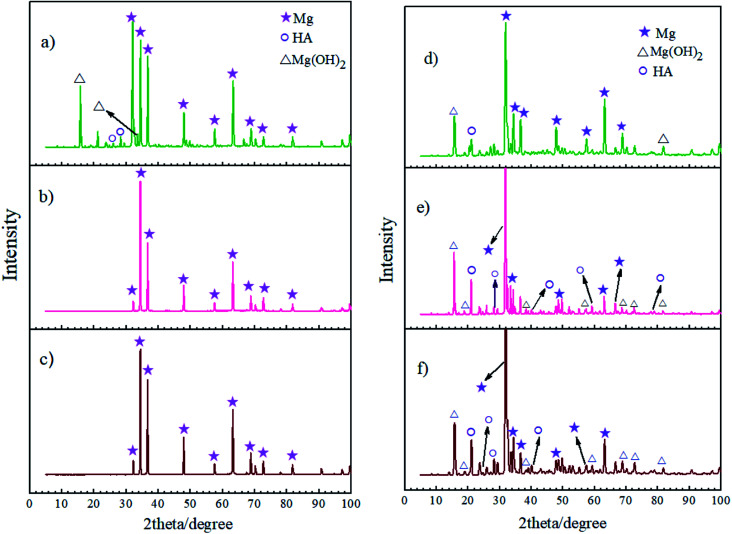
XRD patterns of the corrosion product layers on (a) bare, (b) HA0, (c) HA1000 samples after 7 days of immersion and (d) bare, (e) HA0, (f) HA1000 sample after 21 days of immersion.

Fig. 13 shows the XRD measurements for the corrosion products of the bare, HA0 and HA1000 samples after 7 and 21 days of immersion in SBF. XRD measurements were also carried out on samples before immersion and no new phase emerged as a result of acid pretreatment, this is while EDS analysis proved the high concentration of fluorine on the pretreated samples. Jiang *et al.* reported that after 2 hours of chemical conversion, the layer formed on the substrate was too thin to be detected by XRD analysis. On the other hand the surface composition of the HF pretreated samples seems to be amorphous or nanocrystalline.^56^ In the case of the bare sample, SEM images confirmed the existence of a fibrous corrosion layer (see Fig. 4a), the sharp peaks detected on the bare sample after a week of immersion show that a part of the corrosion product was Mg(OH)_2_. The existence and intensity of Mg(OH)_2_ represents the corrosive degree, the bare sample was the only sample which exhibited this peak after 7 days of immersion, indicating that HA0 and HA1000 possessed better corrosion resistance and that the corrosion product layer was not thick enough to be detected. According to Li *et al.*,^57^ the formation of a Mg(OH)_2_ layer facilitates the hydroxyapatite nucleation by immobilizing calcium on the surface of the substrate. The presence of HA peaks on bare and HA0 after 21 days of immersion seem to be due to the formation of the Mg(OH)_2_ layer as a corrosion product. Worth of notice is the fact that the formation of a corrosion product containing hydroxyapatite promotes bone deposition and hamper metallic ions accessibility into the body fluids. All samples are expected to favor bone formation in the long-run; however, *in vivo* studies should be carried out to provide more information in this case.

### 3.7. *In vitro* cytocompatibility studies

**Fig. 14:**
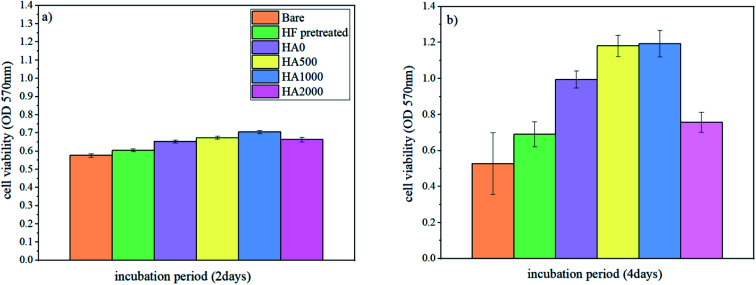
Cell viability/proliferation of osteoblastic cells on different samples (a) after 2 days of incubation, (b) after 4 days of incubation.

It is well known that the fast corrosion process breeds the formation of more magnesium hydroxide, and pH rises above 8, which is an impediment to cell adhesion and proliferation.^54^ As a result, surface modifications like silane coating, which decelerate the corrosion process, are expected to improve cell response. To assess whether the silane coating and HA nanoparticles are influential in cell growth and if they enjoy bioactivity, cell viability/proliferation (MTT assay) was performed for different durations (2 days and 4 days) on the bare, HF pretreated, HA0, HA500, HA1000 and HA2000 samples. Osteoblastic MG-63 cells were seeded on samples to evaluate the effect of silane coating and nanoparticle incorporation on cell growth. Results concerning cell viability/proliferation are shown in Fig. 14. All samples except the bare allowed cell growth over the surface since figures show an increase from day 2 to day 4. There was no staggering difference between cell responses after 2 days of incubation. This meant that at the early stage of immersion when the higher corrosion rate in the bare and HF pretreated samples was almost the same, cell viability did not attain statistical significance. The effect of mere acidic pretreatment was also investigated in this study to tell the effect of silane coating and HF pretreatment apart. *In vivo* tests have previously reported promising bone formation results on the HF treated AZ31 samples. The results of *in vitro* tests in our study were in agreement with findings in ref. 58 as there was an increase in viable cells on the HF pretreated sample after 4 days of incubation. The HA0 sample evinced a higher proliferation and viability rate owing to the controlled corrosion rate. A lower corrosion rate, together with the osteoconductivity property of HA nanoparticles, attained statistical significance in cell viability after 4 days of incubation for HA500 and HA1000. Osteoblasts seeded on HA500 and HA1000 samples exhibited an apparently higher proliferation and a marked decrease in cytotoxicity compared to those of the uncoated and HA0 samples. In this study, HA nanoparticles were found to be advantageous both to corrosion protection and cell proliferation. HA500 did not provide a highly protective corrosion barrier like HA1000. It has been reported that cell proliferation is inversely related to HA nanoparticle size. This is because smaller nanoparticles promote interfacial adhesion of HA nanoparticles to cells and also a greater surface area to volume ratio per HA nanoparticle is provided for cell growth.^42,59,60^ Considering that cell viability results for HA500 are identical to that of HA1000, it can be inferred that a smaller HA nanoparticle size (344.1 nm) in case of HA500 led to a larger number of cells being viable. As a matter of fact, the comparatively weak corrosion protection provided by HA500 is linked with cell death, while a larger number of HA nanoparticles are able to enter into cells and promote cell growth. The promising corrosion protection of HA1000 is accompanied by a larger nanoparticle size (454 nm) and interference in entering into cells. Fewer HA nanoparticles entered into cells in the case of HA1000 and stimulated cell growth, while the satisfying corrosion protection restrained apoptosis. There is no disguising the fact that HA2000 exhibited aggravated cell response in comparison to that of the HA0, which does not contain any bioactive substance to promote cell proliferation (see Fig. 14b). This is presumably due to the weak corrosion barrier provided against aggressive ions together with agglomeration (ineluctable to some extent), which hampers nanoparticle entrance into the cells. On the other hand, it is reported that higher amounts of HA nanoparticles lead to an increase in the amounts of calcium and phosphate in the matrix, which can conduce to apoptosis in osteoblastic cells during bone resorption.^61^ It can be concluded that the proper concentration of HA nanoparticles in silane coatings is favorable due to properties like improved biocompatibility, bioactivity and osteoconductivity,^62–64^ provided that corrosion protection is not endangered.

#### 3.7.1. Adhesion test and FESEM analysis of the MTT samples

**Fig. 15:**
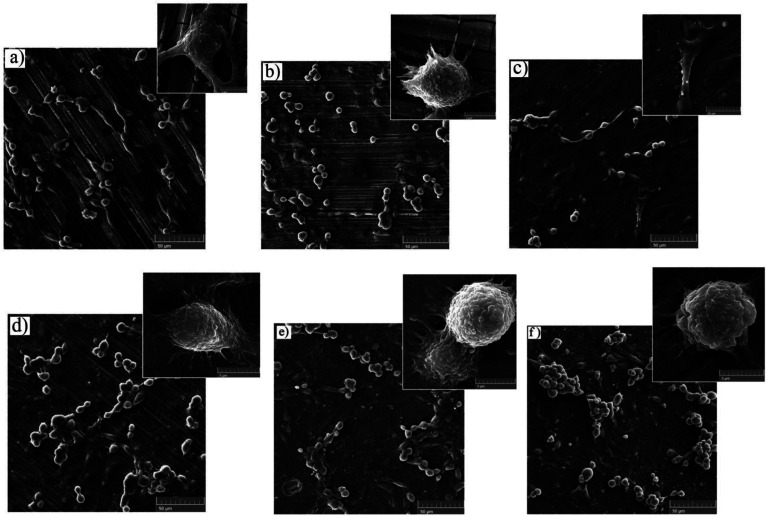
FESEM images of MTT samples conducted on (a) bare, (b) HF pretreated, (c) HA0, (d) HA500, (e) HA1000 and (f) HA2000 after 2 days of incubation. The image above each sample represents the adhesion behavior of the very sample.

**Fig. 16:**
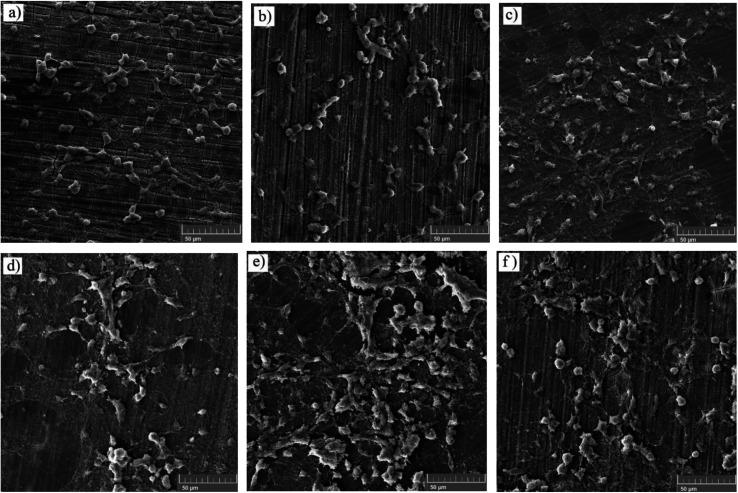
Surface characterization of MTT samples after 4 days of incubation (a) bare, (b) HF pretreated, (c) HA0, (d) HA500, (e) HA1000 and (f) HA2000.

The cell adhesion test was employed to determine the effect of silane coating and modifying it with HA nanoparticles on the biocompatibility of different samples. The inset on the top of each sample in Fig. 15, depicts cell adhesion morphology. In this study, there was no means to count the adhered cells, therefore, the results are discussed only schematically. Cell adhesion is a multifactorial issue, depending on parameters like surface roughness, contact angle, corrosion protection and so forth. In order to achieve the best adherence, higher submicron roughness, lower contact angles and better corrosion protection are favorable. Fig. 15a exhibits a well dispersed and almost interconnected pattern of cell distribution after 2 days of incubation. However, Fig. 16a is replete with cracks which are most likely due to the corrosion process taking place at a high rate during the first days of implantation when no protective film exists. The high degradation rate is conducive to the generation of H_2_ gas pockets, which not only interferes with cell growth but also breeds cell death through high cell osmolality.^65^ After 4 days of incubation, more cracks are visible on the surface of the bare sample, which hamper cell survival and sufficient settlement.^66^ As depicted in the inset above each sample in Fig. 15, adhesion tests reveal detached cells for the bare and HF pretreated samples. The sudden increase in pH value for the bare sample after 2 days of immersion to 8.9 (Fig. 12b) seems to make cell adhesion more difficult since more Mg(OH)_2_ is released. It is interesting to note that cell viability results for the HF pretreated sample were similar to that of the bare sample, suggesting that HF pretreatment did not exacerbate cell proliferation and did not induce additional toxicity. HF pretreatment facilitates cell growth by postponing substrate corrosion and releasing less H_2_ gas. On the other hand, it seems to impede cell adhesion. Though the HF pretreated sample enjoys a rougher surface (*R*_a_ ≈ 22.54 ± 1.365 nm) and better corrosion protection, the higher contact angle (124°) seems to outweigh and culminate in poor cell adhesion. In comparison with the HF pretreated sample, cells on the bare sample were elongated, which is indicative of better cell attachment and healthier cellular morphology, while the pretreated sample shows round and spindle shape cells. Results concerning cell adhesion demonstrate elongated cells and improved adaptation with the underlying substrate for HA0 which is due to the lower pH value (7.46) and fewer gas pockets released as a result of retarded corrosion process. It is well known that hydrophilicity of the surface can ameliorate biocompatibility and promote cell growth.^67^ As discussed in Fig. 2, the contact angle saw a 50 degree decrease for HA0, which facilitates cell adhesion to a great extent. It has been reported that the inorganic part of human bone tissue consists of a nanocrystalline carbonate HA.^18^ This puts the hypothesis forward that HA nanoparticles are probable to provide a more biomimetic environment for osteoblastic cells. MTT results concerning the samples containing 500 and 1000 mg L^−1^ of HA in Fig. 14b revealed a higher growth rate and proliferation of the osteoblastic cells in comparison with those of the others. Cytoplasmic spreading visible in Fig. 16d–f is indicative of the fact that HA nanoparticles have promoted cytocompatibility and biocompatibility of the coatings, which is most probably due to the osteoconductivity property of HA. After 4 days of incubation, densely dispersed osteoblastic cells were visible in Fig. 16e with interconnected cell-to-cell contact, which is highly favorable. This implies the fact that lower contact angles (in comparison to that of the bare) were an aid to cell growth on nanoparticle-containing samples, as well as lower pH values and fewer gas pockets released as a result of the controlled corrosion process. Interconnection between cells and cell contact are improved in Fig. 16e, suggesting that HA nanoparticles were available on the entire surface of HA1000. There was no evidence of cracks or other defects on HA500 and HA1000, indicating that the corrosion process was well controlled and hydrogen evolution did not hamper cell growth. There seem to be a competition between submicron surface roughnesses, lower contact angles and corrosion process. Increasing the surface roughness is reported to be advantageous to cell adhesion and proliferation.^68^ Higher surface roughness can conduce to better cell adhesion, providing that the corrosion process does not take place at a high enough rate to hamper adhesion *via* producing hydrogen gas pockets and cytotoxicity. In the case of the bare, HF pretreated sample and HA2000, higher corrosion rates and contact angles outweigh higher roughness, and consequently, fewer adhered cells are found on the metal surface after 4 days of incubation. Employing 1000 mg L^−1^ of HA nanoparticles seems to provide satisfactory corrosion protection as well as a higher surface roughness which paves the way for cell adhesion. Results concerning HA2000 indicate both higher surface roughness and contact angle but lower corrosion protection. Concerning cell adhesion, the higher contact angle is an impediment and opposes the higher nano range surface roughness, which can be an aid. Fig. 15f reveals some colonized cells, which indicate that the higher contact angle (81°) outweighed and prevented cell elongation in the case of HA2000. These colonized cells did not remain viable after 4 days of incubation, and as observed in Fig. 16f, they lost contact with neighboring cells. This is attributed to poor corrosion protection and the low impedance modulus (1.35 × 10^3^ Ω cm^2^), which lead to pH rise and production of magnesium hydroxide. It can be concluded that higher amounts of HA nanoparticles can result in improved cell growth and proliferation as long as corrosion protection is not aggravated.

## 4. Conclusions

A silane coating containing GPTMS, MTES and TEOS in equal proportions was used to control the corrosion rate of AZ31 magnesium alloy. A set of well-balanced properties including hydrophilicity, low surface roughness and corrosion resistance were attained to a great extent. Coated samples proved corrosion resistance 3 orders higher than that of the bare; nevertheless, samples did not remain corrosion resistant in SBF at 37 ± 1 °C after 7 days of immersion. Hydroxyapatite nanoparticles at different concentrations were added in the hope of enhancing biocompatibility, cell proliferation and prolonging the coating resistance. HA containing samples revealed calcium-and phosphate-enriched corrosion products, which is a crucial feature for bioresorbable implants. Doping 500 mg L^−1^ of hydroxyapatite nanoparticles, as the lowest concentration, did not reveal a notable distinction in corrosion protection compared to that of the HA0. The incorporation of 1000 mg L^−1^ of HA nanoparticles rendered a biofunctional coating which allowed cell proliferation and decelerated the corrosion process simultaneously. The use of HA nanoparticles in an optimized amount could help to sustain the initial corrosion protection achieved after an hour of immersion. For higher amounts of HA (2000 mg L^−1^), agglomeration of nanoparticles was observed, which aggravated corrosion resistance. The results testify that incorporation of 500 mg L^−1^ to 1000 mg L^−1^ provides desirable cell viability with a range of corrosion resistance.

## Conflicts of interest

There are no conflicts to declare.

## Supplementary Material
