# Correction: Silane coatings modified with hydroxyapatite nanoparticles to enhance the biocompatibility and corrosion resistance of a magnesium alloy

**DOI:** 10.1039/d1ra90137k

**Published:** 2021-08-11

**Authors:** Aida Nikbakht, Changiz Dehghanian, Rasoul Parichehr, Kavian Mashayekhi

**Affiliations:** School of Metallurgy and Materials Engineering, College of Engineering, University of Tehran P.O. Box 11155-4563 Tehran Iran aida.nikbakht1995@gmail.com cdehghan@ut.ac.ir rasoulparichehr@gmail.com +98 2188006076 +98 2144500749

## Abstract

Correction for ‘Silane coatings modified with hydroxyapatite nanoparticles to enhance the biocompatibility and corrosion resistance of a magnesium alloy’ by Aida Nikbakht *et al.*, *RSC Adv.*, 2021, **11**, 26127–26144. DOI: 10.1039/D1RA01018B.

The authors regret the omission of one of the authors, Kavian Mashayekhi, from the original manuscript. The corrected author list is as shown above.

The Royal Society of Chemistry apologises for these errors and any consequent inconvenience to authors and readers.

## Supplementary Material

